# New York Academy of Medicine—Section in Orthopædic Surgery

**Published:** 1897-05

**Authors:** 


### Society Notes.

### New York Academy of Medicine.—Section in Orthopaedic Surgery.

MEETING OF MARCH 19, 1897.

A CASE OF WRY-NECK.—Dr. T. H. Myers presented a girl, fifteen years of age who, in last August, after having been heated by hard playing the day before, found her head fixed in a certain position, and that attempts to change the position caused pain. Three or four days subsequently she began to have pain at night in the left side of the neck posteriorly. This has been the condition till the present time. Her general health is good. There is tenderness in the left suboccipital region. At times she supports her head with her hands to relieve pain. There is no headache and no pain on joilting or jarring. There is no rigidity of the superficial muscles on either side of the neck. Flexion and extension of the head are normal. The chin is rotated to the right ten degrees, and the head is inclined a little towards the left shoulder. Attempts to correct this position cause reflex spasm. The first sixth cervical spinous processes are prominent. No prominence can be felt anteriorly on examining the throat. There is no lateral curvature. She had never had rheumatism or any form of joint disease. Leaving out of the question the inception of the disease, almost all the features of the case were those of cervical caries, and Dr. Myers intended to treat the patient for this trouble, but he wished to be able to make a positive diagnosis between this condition and rheumatism of the neck.

Dr. S. Ketch said that it was not always possible to entirely eliminate suspicions of rheumatism in cases which it is necessary to treat as vertebral caries. In the present case he considered the reflex muscular spasm, the limitation of motion and the prominence of the vertebræ as sufficient to determine the diagnosis. He believed the trouble was in the cervical vertebræ not lower than the third, but did not think it was necessarily tubercular.

Dr. R. Whitman thought the trouble was not tubercular. Although the atlas was prominent it was not enough so to have any significance, there was no infiltration and the motion is restricted in some directions but not in all. Occipito-atloid disease impaired the nodding motion and atlo-axoid disease impaired rotation. He thought it probable that the symptoms had followed strain or injury, and that the continuous altitude was causing continuous pain. He would rectify the position and approved the treatment which had been proposed.

Dr. A. B. Judson said that it had sometimes been his experience to refer patients for general treatment, on the ground that the trouble was not local bone disease, and to have clear symptoms of osteitis appear at a subsequent period. The obscurity of the symptoms in osteitis of the cervical vertebræ occasionally made early diagnosis, as in the present case, far from easy. He thought that mechanical treatment was required in this case, and and that it was probably a case of cervical caries.

Dr. V. P. Gibney would rather suggest that this might be a case of irritable spine. He would employ active counter-iritation with fly blisters or the actual cautery and treat the patient as a neurotic. There is often an irregularity of the spinal column even when no pathological condition exists.

A Case of Hip-Disease.—Dr. Myers presented a boy, twelve years of age. When he was seven years old he developed fever and chills, and a large abscess appeared at the right hip with œdema of the limb. An incision showed that five inches of the shaft of the femur were denuded of periosteum. Exsection was not performed, and the long hip-splint had been worn till the present time. The sinuses continued to discharge, and on December 29, 1896, they were explored and found to proceed from cloacæ on the opposite sides of the femur below the trochanter. These were enlarged and sequestra, from half an inch to an inch and a half in length, were removed. On February 3, 1897, the sinuses had closed. The affected limb was three-quarters of an inch longer than the other. There was no pain, and he walked well without the splint. There was very wide motion in flexion and extension. The case showed that conservatism in regard to excising bare bone was necessary and advisable in these cases of osteomyelitis. The periosteum in this case was elevated far beyond the limits of the central necrosis, and an exsection could not have secured a result as good as this.

Dr. Judson said that in its duration and results the case resembles ordinary hip-disease, but a very exceptional feature was the bony lengthening, which was the more remarkable because the trochanter was far above the line.

Dr. L. W. Hubbard recalled but one case of ordinary hip-disease in which careful measurements showed absolutely no bony shortening.

Dr. Whitman recalled a case in which the patient had suffered many years from necrosis of the femur with an inch and a half of lengthening. The bone was broken accidentally, and its non-union was followed by amputation.

Aneurysm Simulating Spinal Disease.—Dr. Ketch related a case in which the patient, a woman thirty-seven years of age, had been advised to seek mechanical treatment for spinal disease. The principal symptoms were radiating pain in the back, loss of flesh, aphonia and occasional dyspnœ. Examination revealed a pulsating tumor at the lower part of the carotid triangle and a decided aneurysmal bruit.

A Specimen of Acetabular Disease.—Dr. Gibney related the case of a boy five years of age, who had had symptoms of left hip-disease for four months. There were marked flexion and adduction, limited motion and an abscess in the gluteal region. After being on an inclined plane with a weight and pulley for a month, there was no improvement, and an incision was made above the trochanter and the capsule was divided. The femoral head appeared to be normal and was not removed. In the acetabulum were broken down bone and erosion, and considerable pus was present. The acetabulum was curetted, the sinuses were drained and the wound was irrigated and packed with iodoform gauze but not sutured. Pneumonia developed two days after the operation and the patient died one month later. Both lungs were found in a state of mottled red and gray hepatization with areas of atelectasis. On section no foci were found in the head or neck of the femur. The articular surface of the head been eroded since the operation. The specimen represented an exaggerated instance of acetabular disease.

